# Mechanochemical Signaling of the Extracellular Matrix in Epithelial-Mesenchymal Transition

**DOI:** 10.3389/fcell.2019.00135

**Published:** 2019-07-19

**Authors:** Lewis E. Scott, Seth H. Weinberg, Christopher A. Lemmon

**Affiliations:** Department of Biomedical Engineering, Virginia Commonwealth University, Richmond, VA, United States

**Keywords:** extracellular matrix (ECM), epithelial-mesenchymal transition (EMT), mechanobiology, cellular signaling, epithelial phenotype

## Abstract

Epithelial-Mesenchymal Transition (EMT) is a critical process in embryonic development in which epithelial cells undergo a transdifferentiation into mesenchymal cells. This process is essential for tissue patterning and organization, and it has also been implicated in a wide array of pathologies. While the intracellular signaling pathways that regulate EMT are well-understood, there is increasing evidence that the mechanical properties and composition of the extracellular matrix (ECM) also play a key role in regulating EMT. In turn, EMT drives changes in the mechanics and composition of the ECM, creating a feedback loop that is tightly regulated in healthy tissues, but is often dysregulated in disease. Here we present a review that summarizes our understanding of how ECM mechanics and composition regulate EMT, and how in turn EMT alters ECM mechanics and composition.

## 1. Introduction

Epithelial-Mesenchymal Transition (EMT) is a transdifferentiation process in which epithelial cells progressively lose key hallmarks of the phenotype, including strong cell-cell contacts, an apicobasal polarity, a cobblestone morphology, and collective migration. Loss of the epithelial phenotype corresponds with an acquisition of a mesenchymal one, with hallmarks including front-back polarity, independent migration, and an elongated cell shape. This process, along with the inverse process, Mesenchymal-Epithelial Transition, is absolutely critical to tissue patterning and organization in developing embryos. However, the past three decades have revealed that dysregulation of EMT is associated with a wide array of pathologies, including fibrotic diseases and cancer. Maintenance of the epithelial phenotype, and subsequent loss of the phenotype during EMT, are highly influenced and controlled by the surrounding extracellular matrix (ECM).

**Figure 1:**
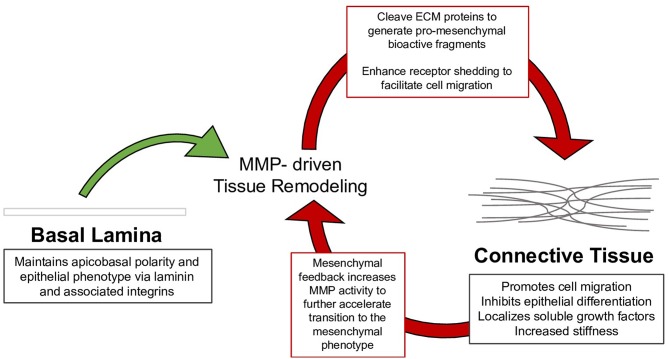
Tissue remodeling of the basal lamina is necessary to maintain healthy epithelium; however, mysregulation of this process drives the assembly of connective tissue, which can in turn facilitate the mesenchymal phenotype. The mesenchymal phenotype further drives tissue remodeling to continue the process in EMT.

**Figure 2:**
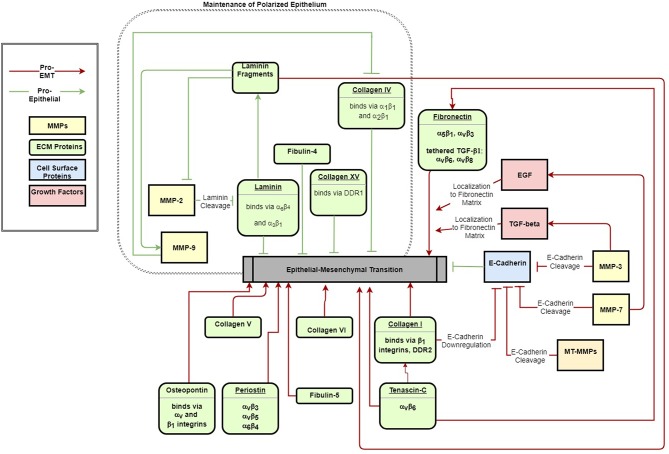
ECM signals both maintain epithelial polarization (green arrows) and drive EMT (red arrows). The schematic indicates major ECM components that have been identified to activate or inhibit EMT.

This review summarizes the myriad interactions between the ECM, epithelial polarization, cell migration, and EMT, and details the following progression of ECM/EMT interactions, illustrated in Figure 1: the composition of the basal lamina drives epithelial differentiation; during routine tissue remodeling, MMPs activate migration through structural changes to the ECM, release of bioactive fragments, and shedding of cell-bound receptors. Each of these events, intended to maintain healthy tissue, prevents an avenue for dysregulation. Conversion to a fibrillar matrix upregulates mesenchymal transcription factors and represses epithelial markers to drive EMT. In a feed-forward process, EMT in turn upregulates many of these fibrillar ECM components. In addition to presenting an ECM composition which facilitates the mesenchymal phenotype, fibrillar ECM also drives changes in tissue stiffness, which further add to epithelial repression and mesenchymal differentiation. Finally, a host of matricellular proteins modify the ECM response to further regulate EMT. Pathways involved in these processes are summarized in Figure 2.

## 2. The Basal Lamina and Epithelial Differentiation

In a polarized epithelium, attachment to the ECM orients the apicobasal axis by defining the basal surface (Morrissey and Sherwood, 2015). Basement membranes are thin, dense specialized ECM that self-assemble at the basal surface of the epithelial sheet as a structural scaffolding for epithelial attachment (McKee et al., 2007). The innermost layers of the basement membrane are the cell-adherent layers, and include the laminin-rich *lamina lucida* and the collagen IV-rich *lamina densa*. Together these layers, joined by glycoprotein nidogen and heparan sulfate proteoglycans perlecan, agrin, and collagen VII, comprise a supramolecular, reticular structure known as the basal lamina (Hohenester and Yurchenco, 2013).

### 2.1. Basal Lamina Establishes the Epithelial Apicobasal Polarity

Laminins are a family of glycoproteins consisting of an α-β-γ heterotrimer that forms three separate short arms (laminin-α_A_β_B_γ_G_) and an extended triple helical coiled-coil long arm arranged in a cross-like confirmation. Overlapping lateral interactions along the triple-helical domain and N- and C-terminal domains provides stability to the collagen IV network (Khoshnoodi et al., 2008). Collagen VII (Rousselle et al., 1997) and microfibril collagen VI (Yurchenco, 2011) stabilize the *lamina densa* and link it to the reticular lamina. Mammary epithelia exhibit normal physiological function when in contact with laminin, but lose this when exposed to fibrillar matrix components, such as collagen I and fibronectin. Developmental studies indicate that laminin not only maintains epithelial differentiation but is also the progenitor of the basement membrane and epithelial polarization during gastrulation (reviewed in Li et al., 2003), suggesting laminin contributes to epithelial differentiation rather than mesenchymal suppression (Zutter et al., 1995; Ramirez et al., 2011).

Laminins form a cell-adherent sheet-like matrix at the cell surface through interactions with its receptors. The laminin G domain located at the α-chain C-terminus of the long arm, together with adjacent coiled-coil domains, mediates cell attachment to integrin, dystroglycan, sulfated glycolipids, and heparan sulfate chains (Yurchenco and Patton, 2009). While laminin deposition and organization at the cell surface requires attachment to dystroglycan (Henry and Campbell, 1998; Henry et al., 2001) and β_1_ integrin (Li et al., 2002), assembly of laminin into sheets occurs spontaneously (Hamill et al., 2009). The laminin specific integrin receptors α_3_β_1_ and α_6_β_4_ additionally contribute to the integrity of the epithelium by reinforcing cell-cell junctions: integrin α_3_β_1_ is localized to the cytoplasmic plaque of cell-cell junctions, where it forms a complex with α-actinin and links the subcortical actin network to the catenin complex of cell-cell junctions (Wang et al., 1999); integrin α_6_β_4_ is localized to a multiprotein complex known as the hemidesmosome, which anchors the cytoskeleton to the basal lamina, provides attachment for intermediate filaments, and confers tensile strength to the epithelium (Borradori and Sonnenberg, 1999). Though not necessary for assembly, laminin-integrin ligation does stimulate small GTPases to restructure the cytoskeleton to support epithelial polarization (reviewed in Lee and Streuli, 2014). Integrin receptors for collagen and laminin were further shown to have a dynamic role in sensing the basal cue through distinct GTPase activity (Myllymäki et al., 2011): while α_6_β_4_ (along with collagen receptor α_2_β_1_) activates Rac-mediated actin cytoskeleton reorganization that guides apicobasal polarity, α_3_β_1_ stimulates Cdc42-mediated microtubule rearrangement, which guides polarity complexes to the respective apical and basolateral domain. These contacts, together with a basement membrane linked to connective tissue, form a cohesive and mechanically coupled structure.

### 2.2. Basal Lamina Components Regulate EMT

Despite its role in maintaining epithelial differentiation, elements of the basal lamina can also promote EMT and tumor growth. For example, laminin receptors α_3_β_1_ and α_6_β_4_ have been implicated in EMT and cancer progression. In a study of keratinocytes, α_3_β_1_ and α_6_β_4_ integrin ligation was sufficient to form tumors despite abnormal cell attachment (Waterman et al., 2007). Deletion of the α_3_β_1_ gene itga3 deletion prevented tumor initiation with an associated decrease in downstream activation of EMT-linked FAK, Rac1, MAPK, and JNK pathways (Cagnet et al., 2014). Studies of alveolar epithelium (Kim et al., 2009a,b) and hepatocellular carcinoma (Giannelli et al., 2005) demonstrate cooperative activity between α_3_β_1_ and transforming growth factor β_1_ (TGF-β1) to suppress the epithelial phenotype. Colocalization and endocytosis of α_3_β_1_ with the TGF-β receptor type I (TGFβRI) receptor led to phosphorylation of Smad2 and of β-catenin on Y654, resulting in formation of a pSmad2-Y654-β-catenin complex, though it is unclear how this complex suppresses the epithelial phenotype. A separate study in immortalized mouse keratinocytes demonstrated that α_3_β_1_-TGF-β1 cooperativity induces tissue remodeling of the basal lamina by stimulating MMP9 (Sugiura and Berditchevski, 1999; Morini et al., 2000) and induces epithelial transcriptional suppressors, Snail and Slug (Zhang et al., 2003). Hepatocellular carcinomas overexpressing α_6_β_4_ exhibit aberrant cell proliferation and invasion associated with downregulation of the epithelial phenotype by PI3K/Akt signaling dependent upregulation of Slug (Li et al., 2017).

In addition, collagen IV, which is a primary component of the basement membrane, can suppress epithelial differentiation and induce expression of the EMT transcription factors Snail and Slug. Hepatocellular carcinomas express collagen IV receptors α_1_β_1_ and α_2_β_1_ to facilitate local invasion across the basal lamina and lamina reticularis (Yang et al., 2003). In mammary epithelial cells, collagen IV induces epithelial repressors Snail and Slug by upstream FAK/ERK signaling and NFκB activation (Espinosa Neira and Salazar, 2012).

Another component of the basement membrane is Collagen XV, which forms an unusual supramolecular structure resembling a figure-of-eight/pretzel configuration (Myers et al., 2007). This structure links banded fibrils in the basement membrane (Amenta et al., 2005) to provide tensile strength and join the basement membrane to connective tissue. Collagen receptor discoidin domain receptor 1 (DDR1) is distributed to the lateral membrane of epithelium to stabilize cell-cell junctions. This suppresses Cdc42 activity (Wang et al., 2009) and prevents α_2_β_1_-DDR1 cooperative activation of migration. In pancreatic adenocarcinoma cells, collagen XV was shown to stabilize cell-cell junctions by DDR1, suggesting antagonistic EMT potential (Clementz et al., 2013).

## 3. Tissue Remodeling and EMT

Tissue remodeling in development (Brauer, 2006) and tissue repair (Page-McCaw et al., 2007; Rodríguez et al., 2010) requires fine spatiotemporal control over ECM degradation, which is often dysregulated in fibrosis (Giannandrea and Parks, 2014; Craig et al., 2015; Pardo et al., 2016) and cancer progression (Nabeshima et al., 2002; Têtu et al., 2006; Kessenbrock et al., 2010; Deryugina and Quigley, 2015). Through ECM and growth factor proteolysis, matrix metalloproteinases (MMPs) modify the molecular and mechanical characteristics of the extracellular microenvironment to facilitate cellular migration (Sternlicht and Werb, 2001). MMPs consist of a catalytic domain, an autoinhibitory prodomain, and hemopexin domain. The six classifications of MMPs–collagenase, gelatinase, stromelysin, matrilysin, membrane-type and non-classified MMPs–delineate substrate specificity that is further tied to cellular and extracellular localization (Nagase et al., 2006). Functionally, MMPs regulate motility motifs by proteolytically processing ECM components and its sequestered latent signals, as well as membrane receptor docking and shedding (Sternlicht and Werb, 2001). Receptor docking spatially confines remodeling, whereas proteolytic byproducts act as soluble signals to engage feedback loops that temporally maintain ECM degradation.

### 3.1. Growth Factors and Bioactive Fragments Activate EMT

Beyond the structural effects on ECM, MMP proteolytic processing of the basal lamina produces bioactive fragments (Horejs, 2016), many of which regulate angiogenesis (Xu et al., 2001) and migration (Horejs et al., 2014) in a paracrine fashion. For example: Collagen IV fragment α_5_(IV) binds collagen receptor DDR1, preventing distribution to cell-cell junctions, and activates ERK (Xiao et al., 2015), a downstream signal of TGF-β1-induced EMT (Xie et al., 2004; Buonato and Lazzara, 2014), and a laminin-111 β-chain fragment competitively binds α_3_β_1_ integrin to upregulate mesenchymal markers and switch gelatinase A (MMP2) production in the inner lamina lucida to gelatinase B (MMP9) in the outer lamina densa and reticular lamina.

Additionally, MMPs dock with cell adhesion receptors, facilitating proteolytic activation of latent signaling molecules sequestered within the ECM and inducing survival and migratory signaling pathways (Illman et al., 2006; Chaturvedi and Hass, 2011; Mori et al., 2013). Redistribution of MMPs to the migratory front mediates focal proteolysis that is spatially confined to the invadopodia (reviewed in Jacob and Prekeris, 2015): gelatinases (Yu and Stamenkovic, 2000), stromelysin (MMP3) (Maeda et al., 2002), and membrane type (MT)-MMPs (Mu et al., 2002) each proteolytically activate latent form of TGF-β1, and the subsequent TGF-β1 signaling upregulates gelatinases, creating a self-sustaining loop of matrix remodeling (Krstic and Santibanez, 2014). To this point, knockdown of MMP9 abrogated mesenchymal markers and inhibited TGF-β1-induced invasion and migration in a study of esophageal squamous cell carcinoma (Bai et al., 2017).

### 3.2. Receptor Shedding Enhances Mobility

Another means by which tissue remodeling drives EMT is through MMP-mediated receptor shedding. MT-MMP (Rozanov et al., 2004), MMP3 (Yamashita et al., 2011), MMP9, and matrilysin (MMP7) (McGuire et al., 2003) localize to adherens junctions to shed the E-cadherin ectodomain, producing a soluble fragment frequently increased in the serum of cancer patients (Repetto et al., 2014). The 80 kDa ectodomain fragment acts as a paracrine/autocrine signal that reduces cell aggregation by competitive homophilic binding with E-cadherin (Noe et al., 2001) and promotes MMP production via EGFR (David and Rajasekaran, 2012). MMP3 additionally cleaves E-cadherin, which specifically activates Rac1 splice variant Rac1b that in turn activates ROS/Snail (Radisky et al., 2005).

### 3.3. EMT Induces MMP Production

Soluble factors released during tissue remodeling can induce EMT, which in turn stimulates MMP production to sustain remodeling of the ECM for migration (reviewed in Gilles et al., 2013 and Bonnans et al., 2014). Activation of EMT transcription factors corresponds with an increased secretion of gelatinases in human mammary breast cancer epithelium (Octavio et al., 2015) and oral squamous cell carcinoma (Qiao et al., 2010). Specifically, Slug directly enhances MMP1 transcription in breast cancer cells (Shen et al., 2017a) as well as MMP9 in oral squamous cell carcinoma (Joseph et al., 2009), and Snail upregulates MMP9 in MDCKs (Jordà et al., 2005). Additionally, Kruppel-like factor 8 (KLF8), a downstream transcription factor of TGF-β1 signaling, upregulates MMP9 (Wang et al., 2011) and MT1-MMP, both directly (by activating MT1-MMP promoter) and indirectly (through β-catenin nuclear translocation and TCF1 upregulation) (Lu et al., 2014). Proteolytically activated growth factors induce these transcription factors, resulting in a positive feedback loop. TGF-β1-dependent EMT triggers gelatinases in oral squamous cell carcinoma by upregulated Snail (Qiao et al., 2010) and MMP10 in keratinocytes (Wilkins-Port and Higgins, 2007). Wnt stimulates MMP production that amplifies tissue remodeling and cell mobility. For example, MT3-MMP is a soluble and membrane bound proteinase that cleaves pro-MMP2 and is associated with progression of hepatocellular (Wu et al., 2007) and colorectal (Shen et al., 2017b) carcinomas.

## 4. Connective Tissue and Mesenchymal Differentiation

### 4.1. Fibrous Matrix Promotes Attachment and Migration

Unlike the basal lamina, the ECM of connective tissue is fibrillar and primarily deposited by activated fibroblasts (Hosper et al., 2013). Assembly of fibrillar matrix on the basement membrane plays a significant role in repressing the epithelial phenotype and inducing EMT. The primary component of connective tissue, fibrillar collagen, is crudely aligned parallel to the basal lamina to provide mechanical support by resisting tensile forces. Non-fibrillar collagens, proteoglycans, and glycoproteins organize the fibrillar matrix and link the connective tissue to the basement membrane. Fibronectin, a 230–270 kDa fibrous homodimer glycoprotein, provides a scaffold for cell attachment and *de novo* fibrillogenesis (Hynes, 1982). Secreted as a soluble dimer, maturation of the fibronectin matrix requires integrin attachment and cell contractility for polymerization into an insoluble fibrillar matrix (Mao and Schwarzbauer, 2005; Weinberg et al., 2017). Integrin binding facilitates fibronectin stretch, which exposes additional binding sites for ECM deposition and growth factor binding (Singh et al., 2010). As a scaffold for *de novo* ECM assembly, polymerized fibronectin provides binding sites for collagenous matrix deposition (Sottile et al., 2002). However, in a negative feedback loop that halts *de novo* ECM synthesis, maturation of collagenous matrix stabilizes polymerized fibronectin but shields it from cell contractility (Kubow et al., 2015).

Integrin and syndecan receptors link the fibronectin matrix to the cytoskeleton and signaling pathways during migration (Elfenbein and Simons, 2013). Integrins α_5_β_1_ and α_v_β_3_ dynamically bind to the fibronectin matrix to extend pseudopodia and form contractile filaments via small GTPases (Morgan et al., 2009). Fibronectin binding organizes nascent cell contacts into stable focal adhesions that activate the conventional integrin signaling pathways associated with growth and motility. Antagonistic α_5_β_1_ recycling to the migratory front maintains cell-matrix adhesion whereas α_v_β_3_ is recycled to the migratory front to form nascent focal adhesions to fibronectin and stimulate Rac-mediated cytoskeletal rearrangement (White et al., 2007; Lawson and Burridge, 2014). The sustained β_1_ integrin signaling stimulates Rho-mediated contractility as the nascent adhesion moves peripherally to centrally and matures to a fibrillar adhesion near the center and posterior of the cell. Syndecan, as a co-receptor for cell attachment, facilitates integrin clustering into focal adhesions (Woods et al., 2000); mediates small GTPase activity (Bass et al., 2007; Brooks et al., 2012); regulates extracellular matrix assembly (Klass et al., 2000); and, specifically, binds to the fibronectin type III_12−14_ heparin-binding domain adjacent to α_5_β_1_ to facilitate cell attachment (Tumova et al., 2000).

Collagens form the bulk of connective tissue, consisting of a family of 28 members classified as either fibril forming or network forming (Ricard-Blum, 2011). Three polypeptide α-chains of repeating gly-X-Y sequence and interrupting non-collagenous domains form a helical trimer, which is stabilized by glycine, proline and hydroxyproline, hydrogen bonds, and electrostatic interactions, allowing collagen to assemble unique structures and participate in biological activity (Shoulders and Raines, 2009). Collagen receptors β_1_ integrin heterodimers (α_1_, α_2_, α_10_, and α_11_) and DDRs relay adhesion signals at the cell-cell and cell-matrix interface to exert transcriptional regulation of migration and survival signaling pathways (Boraschi-Diaz et al., 2017). DDR1 stabilizes adherens junctions in the stable epithelium but redistributes to cell-matrix focal adhesions to regulate motility (Huang et al., 2009; Chen et al., 2016). Minor collagens, elastic fibers, and proteoglycans support fibrillar collagens to confer structural integrity to connective tissues (Frantz et al., 2010). Collagen V associates with fibrillar collagens I and III to regulate assembly, and collagen VI to stabilize the ECM (Mak et al., 2016). Collagen VI forms beaded filament networks at the stroma-basement membrane interface and organizes three-dimensional tissue architecture by linking connective tissue with collagen IV (Kuo et al., 1997) and laminin (Cescon et al., 2015) of the basal lamina.

Proteoglycans, owing largely to their glycosaminoglycan polysaccharide chains, hydrate tissues and provide mechanical support by resisting compression. Hyaluronan, a glycosaminoglycan synthesized by hyaluronan synthase (HAS), is secreted in a high molecular weight form consisting of N-acetylglucosamine and glucuronic acid repeats. The hyaluronan receptor CD44 activates Src, Rho, and Ras signaling pathways to alter cytoskeletal arrangement and promote motility (Turley et al., 2002). CD44 in particular regulates mobility by interacting with and stimulating production of MMPs. For example, hyaluronan-CD44 binding stimulates gelatinase secretion to regulate cancer invasion (Park et al., 2002; Zhang et al., 2002; Guo et al., 2012) and MMP9 docks with CD44 to remodel the ECM at the invasive front (Peng et al., 2007). Thus, hyaluronan and its receptors mediate mobility through connective tissue.

### 4.2. Fibrillar Matrix Inhibits Epithelial Differentiation

Dysregulation of fibrillar matrix is widely implicated in fibrosis (Karsdal et al., 2017) and cancer progression (Fang et al., 2014; Kaushik et al., 2016; Wang and Hielscher, 2017), owing to the suppression of epithelial differentiation (Shintani et al., 2008b). As a downstream target of Wnt (Gradl et al., 1999) and TGF-β1 (Kolosova et al., 2011) signaling, fibronectin is a marker of mesenchymal differentiation (Petrini et al., 2016). Fibronectin accumulation at cleft-forming sites during salivary gland and lung branching morphogenesis induces Slug to suppress the epithelial phenotype (Onodera et al., 2010). At the invadopodia, α_v_β_3_ ligation induces Slug expression (Knowles et al., 2013) and α_5_β_1_ integrin stimulates Rho-mediated contractility (Mierke et al., 2011).

Fibrillar collagens, acting through canonical β_1_ integrin/FAK/Src signaling, suppress epithelial differentiation at the transcriptional level and disrupt the cadherin complex to enhance cell mobility (reviewed in Imamichi and Menke, 2007). In ovarian and prostate cancer cells, collagen-β_1_ binding alters E-cadherin expression through both PI3K- (Cheng and Leung, 2011) and Src-dependent mechanisms (Menke et al., 2001). Collagen I promotes Snail and LEF1 through ILK-dependent activation of NF-κB and inhibition of GSK3β, which drives transcriptional activation of Snail (Medici and Nawshad, 2010). In a separate study of pancreatic carcinoma cells, DDR1 and β1 integrin concomitant activation converges on JNK signaling to increase expression of N-cadherin (Shintani et al., 2008a), which is a marker of EMT. However, H-Ras-induced Zeb reduces DDR1 expression in mammary epithelial cells, suggesting a negative feedback loop during EMT (Koh et al., 2015).

Given its role in maintaining adherens junctions, collagen-DDR signaling may indicate a switch from cell-cell to cell-matrix adhesion. Switching from epithelial-associated to mesenchymal-associated DDR drives mesenchymal differentiation by activating and stabilizing EMT transcription factors Snail and Zeb, and by inducing gelatinases to promote invasion (reviewed in Rammal et al., 2016). Collagen I-DDR2 ligation induces invasion of metastatic mammary epithelium *in vivo* and *in vitro* by activating Src/ERK signaling to phosphorylate Snail, which facilitates stabilization and nuclear translocation (Zhang et al., 2013). Similarly, in human renal proximal tube epithelial cells, increased DDR2 expression by TGF-β1 suppresses epithelial differentiation via NF-κB and LEF-1 activation (Walsh et al., 2011). As a result, fibrillar collagens, specifically type I and III, are potent suppressors of epithelial differentiation, reducing cell aggregation by transcriptional regulation and disrupting junctions.

Reinforcing other collagenous components of connective tissue also regulates EMT. As a gene target of TGF-β1, aberrant collagen V expression is characteristic of fibrosis and cancer in various tissues (Mak et al., 2016). Overexpression of collagen V in idiopathic pulmonary fibrosis is driven via both a Twist-dependent mechanism (Lei et al., 2016) and STIM1, an endoplasmic reticulum Ca^2+^ sensor that regulates Ca^2+^ influx and promotes invasion of colorectal cancer (Zhang et al., 2015, 2017). Co-immunoprecipitation identified TGF-β1 and MMP2 as binding partners of collagen V (Symoens et al., 2011), suggesting collagen V sequesters TGF-β1 and MMP2 in the ECM to restrict bioavailability and exert spatiotemporal control over tissue remodeling. Collagen VI and its non-integrin receptor NG2, a transmembrane chondroitin sulfate proteoglycan, are highly expressed in tumors (reviewed in Chen et al., 2013) and at the invasive front (Park and Scherer, 2012) where they synergize with canonical TGF-β1 signaling to enhance EMT. NG2 stabilizes cytoplasmic β-catenin and phosphorylates GSK3β downstream of β_1_ integrin-induced Akt (Chekenya et al., 2008), resulting in activation of TCF/LEF and nuclear accumulation of β-catenin (Iyengar et al., 2005).

Hyaluronan is another key component of the connective tissue ECM that stimulates EMT. CD44 mediates hyaluronan-induced invasion through interactions with MMPs that result in focal proteolysis and CD44 cleavage: CD44 ligation localizes MMP9 to the migratory front where it remodels the ECM and cleaves CD44 (Chetty et al., 2012). MT1-MMP additionally localizes to invadopodia where it sheds the CD44 ectodomain to promote motility (Kajita et al., 2001). Aberrant expression of hyaluronan is observed in the tumor stroma of breast, lung, and prostate cancer; urine of bladder cancer; and serum of ovarian, head and neck, and prostate cancer(reviewed in Chanmee et al., 2016) where it primes the cell and microenvironment for invasion(reviewed in Toole, 2004). Hyaluronan induces LOX-mediated matrix stiffening, FAK/Erk signaling, and Twist downstream of CD44 in the progression of breast carcinoma (El-Haibi et al., 2012). In addition, downstream PI3K/Akt and GSK3β phosphorylation results in β-catenin nuclear translocation (Zoltan-Jones et al., 2003), suggesting hyaluronan regulates the epithelial phenotype by inhibiting the catenin complex formation. Despite accumulation in the tumor microenvironment and association with aggressiveness, neither hyaluronan nor CD44 are required to induce mesenchymal transcriptional promoters. Instead, hyaluronan synthase 2 (HAS2) regulates TGF-β1-induced EMT in normal murine mammary gland cells independent of hyaluronan synthesizing activity (Porsch et al., 2013). Knockdown of HAS2 resulted in decreased TGF-β1-induced migration, suggesting a potential role downstream of TGF-β1 signaling. Thus, hyaluronan, along with its synthase and CD44 receptor, not only create a microenvironment to support migration, but also actively suppress epithelial proteins to promote EMT.

### 4.3. Connective Tissue Regulates Soluble Factor Localization

Just as in the basal lamina, proteoglycans of connective tissue sequester soluble factors as a means to regulate bioavailability or to spatially confine activation. Neighboring the primary cell attachment domain of fibronectin, a growth factor binding domain localizes growth factor signaling near the cell attachment for simultaneous activation of signaling pathways that promote survival and migration (Vega and Schwarzbauer, 2016). One example of this in EMT is the latent TGF-β-binding protein (LTBP), which binds to fibronectin at the type III12-14 repeat and sequesters TGF-β1 in a conformationally latent form until mechanically or proteolytically activated (Zilberberg et al., 2012; Robertson et al., 2015). Confining TGF-β1 to the ECM in this latent form allows for spatial and temporal control over TGF-β1 activation (Rifkin, 2005). In the ECM-bound latent form, TGF-β1 is activated through cell contractility or proteolytic cleavage. The α_v_ integrins, namely α_v_β_6_ and α_v_β_8_, bind to the RGD sequences in fibronectin and latent TGF-β complex LAP to conformationally alter LAP and activate TGF-β1 (Mamuya and Duncan, 2012).

Taken together, conventional integrin signaling and growth factor availability regulation represent two distinct but interacting mechanisms by which fibrillar ECM regulates EMT (Hynes, 2009). Previous studies of mammary breast epithelium suggest fibronectin, but not laminin, is necessary for TGF-β1-induced EMT, likely due to binding the fibronectin receptor α_5_β_1_ integrin and latent TGF-β1 localization (Park and Schwarzbauer, 2014; Griggs et al., 2017). Fibronectin receptor α_v_β_3_ integrin has also been shown to phosphorylate TGFβRII at Y284 to activate p38/MAPK signaling, separately from canonical Smad signaling, and promote tumor invasion (Galliher and Schiemann, 2007).

### 4.4. Tissue Mechanics Inhibit Epithelial Differentiation

In addition to the compositional aspects of ECM-EMT regulation, the mechanical properties of the ECM play a key role in regulating epithelial differentiation and EMT. Prior studies have demonstrated that induction of EMT is dependent on the mechanical properties of the underlying tissue; *in vitro*, TGF-β induces EMT on surfaces with a high elastic modulus, but induces apoptosis on surfaces with a lower elastic modulus (Leight et al., 2012). Inherent tension within a tissue also induces EMT; areas of higher stress within a colony of epithelial cells correlates with EMT, while ares of lower stress maintain the epithelial phenotype (Gomez et al., 2010). As such, the mechanics of the ECM play a critical role in driving EMT.

Mechanical coupling between a cell and its environment allows for rapid signal transduction and propagation across the tissue. Mechanotransducers, the cell adhesion receptors and focal adhesion proteins that link the ECM to the cytoskeleton and intracellular signaling cascades, reorient the cytoskeleton to mitigate anisotropic tension (Tseng et al., 2012). Expression of the EMT marker vimentin, which is assembled into intermediate filaments, has been shown to enhance mechanotransduction (Conway and Schwartz, 2014) and mediate growth of focal adhesions (Liu et al., 2015); similarly, organization of the microtubule network regulates both contractile and propulsive force generation (Kent and Lele, 2016), which facilitates migration and protrusion during EMT (Whipple et al., 2010; Gu et al., 2016). Although connective tissue lacks the inherent organization of self-assembled basal lamina, tension pulls the fibers into alignment, parallel to the direction of applied force. The fibrillar components stiffen the ECM and shift cell adhesion and cytoskeletal arrangement toward a migratory scheme.

Deposition and organization of the ECM is sensitive to substrate stiffness (Eisenberg et al., 2011). Fibronectin assembly correlates with substrate stiffness (Williams et al., 2008; Scott et al., 2015), which is not the case with self-assembled laminin in basal lamina. A proposed mechanism suggests stretch of fibronectin type III repeats exposes additional growth factor and ECM binding sites (Weinberg et al., 2017). Stiffer matrices promote greater fibrillogenesis, which in turn facilitates excessive matrix deposition, growth factor tethering, and further stiffening of the ECM (Kubow et al., 2015). It has been proposed that one possible role for collagen in fibronectin deposition is to provide a rigid collagen network that increases tension in the matrix to facilitate fibronectin assembly (Singh et al., 2010). This mechanism would be similar to the tensional effects of cell-cell interactions on fibronectin assembly in Xenopus embryos (Dzamba et al., 2009).

Mechanical feedback at cell-matrix interfaces is an important regulator of EMT (Le Bras et al., 2012). In addition to matrix deposition, the effects of matrix stiffening may enhance signaling of tethered growth factors. In alveolar epithelial cells, fibronectin facilitates stiffness-dependent EMT induced by TGF-β. The requirement for integrin α_v_ that binds both fibronectin and the TGF-β1 complex suggests cell contractility mediates the substrate stiffness response to TGF-β1-induced EMT (Markowski et al., 2012; Brown et al., 2013). The α_v_ integrin activates latent TGF-β, which in turn induces LOX production (Sethi et al., 2011; Voloshenyuk et al., 2011), crosslinks collagen, and stiffens the ECM. Together, these findings suggest integrin receptors mediate the stiffness trends of fibronectin-TGF-β1 activation of EMT in a contractility-dependent manner. In NMuMG cells, matrix rigidity regulates the switch between TGF-β1 induced apoptosis and EMT via FAK/PI3K/Akt signaling (Leight et al., 2012). Additionally, ECM stiffness plays a role in nuclear signaling: nuclear localization of Twist is observed with increased substrate stiffness due to β_1_ integrin activation (Wei et al., 2015); Yes-associated protein (YAP) and transcriptional co-activator with PDZ binding motif (TAZ) are nuclear relays of matrix rigidity downstream of Rho GTPase (Dupont et al., 2011); and nuclear translocation of Hippo effectors YAP and TAZ regulate a RhoA-dependent feedforward mechanism (Calvo et al., 2013) of cell spreading that controls focal adhesion assembly (Nardone et al., 2017). Mechanical feedback also drives rearrangement of cytoskeletal components in EMT, which in turn, can drive EMT (reviewed in Sun et al., 2015).

Similarly, mechanical signaling facilitates events at the cell-cell junction. Cadherin complexes transduce tension between adjacent cells during early epithelialization (Huang et al., 2012). Adherens junctions recruit actin to reinforce adhesion on substrates of high traction stress and strain (Collins et al., 2017). Tensile forces that are exerted on cadherin complexes result in the unfolding of α-catenin to reveal cryptic vinculin-binding sites, which nucleate polymerization of new actin microfilaments (Yonemura et al., 2010). G-actin depletion from the cytoplasmic pool stimulates nuclear accumulation of myocardin-related transcription factor (MRTF)-A, a regulator of actin alignment, to promote cellular contraction (O'Connor et al., 2015). MRTF-A activity also regulates myogenic features (Seifert and Posern, 2017), and is a downstream mediator of TGF-β1-induced EMT (Gomez et al., 2010).

## 5. The Functional Role of ECM Matricellular Proteins

In addition to the specific ECM components discussed here, there are a host of non-structural matricellular proteins that play a role in EMT signaling. A functional role is observed both in the extracellular environment, by soluble signal sequestering and receptor binding, and the intracellular environment, by orchestrating cytoskeletal arrangement or activating mesenchymal transcriptional promoters. Matricellular proteins also are involved in the cellular mechanical response, as mechanosensor integrins are receptors for many of the proteins.

### 5.1. Osteopontin

Osteopontin is a 44 kDa aspartic acid rich, N-linked glycosylated phosphoprotein with widespread expression across tissues. The osteopontin receptors, integrins α_v_ and β_1_, and CD44, stimulate tissue remodeling, inflammation, and biomineralization signaling pathways. Due to its role in tissue remodeling and inflammation, aberrant expression is frequently associated with fibrosis and cancer (reviewed in Zhao et al., 2018), owing to induction of the mesenchymal pathways PI3K and MAPK and canonical transcriptional regulators Twist, Snail, and Zeb (Kothari et al., 2016). Together, these suggest osteopontin potently elicits EMT, further supported by the interaction with TGF-β1 to activate fibroblasts for tissue remodeling.

### 5.2. SPARC

Secreted protein acidic and rich in cysteine (SPARC) is a glycoprotein consisting of Ca^2+^ binding domains and a disulfide, copper binding follistatin domain. SPARC influences collagen I and IV organization, and sequesters growth factors to inhibit receptor binding (reviewed in Murphy-Ullrich and Sage, 2014). Overexpression is observed in a number of cancers (Arnold and Brekken, 2009) and is accompanied by increased expression of mesenchymal markers. Epidermal melanocytes exhibit E-cadherin suppression as a result of increased expression of Snail (Robert et al., 2006) and Slug dependent on PI3K/Akt signaling (Fenouille et al., 2012).

### 5.3. Periostin

Periostin consists of four alternatively spliced isoforms that consist of a small cysteine-rich module, fasciclin-like domains, and a hydrophilic carboxy terminal. The fasciclin-like domains mediate cell adhesion, while the hydrophilic carboxy terminal binds ECM proteins, such as collagen, fibronectin, tenascin-C, and heparin. Periostin is both a marker and promoter of EMT, which contributes to the progression of a number of tumor types (Morra and Moch, 2011). Expression is regulated by EMT transcription factor Twist and growth factors TGF-β1, BMP-2, PDGF, bFGF, TNFα, IL-4, IL-13, angiotensin II, and oncostatin (González-González and Alonso, 2018). Studies indicate periostin elicits a pronounced EMT response by downregulating miR-381, a Twist and Snail repressor, via MAPK (Hu et al., 2017).

### 5.4. Tenascin

Tenascins are a four member family of ECM glycoproteins consisting of N-terminal heptad repeats, EGF-like repeats, fibronectin type III repeats, and a C-terminal fibrinogen-like globular domain (Hsia and Schwarzbauer, 2005). Tenascin-C assembles into hexamers that regulate integrin, proteoglycan, and immunoglobulin binding to the ECM, primarily through interactions with fibronectin. FAK phosphorylation and Src activity together with β-catenin nuclear localization suggest tenascin induces EMT by integrin binding (Yoshida et al., 2015). TGF-β1 stimulates significantly greater tenascin-C secretion for mesenchymal cells relative to epithelial cells via ERK/MAPK but not PI3K signaling (Maschler et al., 2004).

### 5.5. Fibulin

Fibulins are a five isoform family of alternatively spliced anaphylatoxin-like repeats, calcium binding EGF-like repeats, and a fibulin-type carboxyl terminus. Calcium ligation confers structural stability to fibulin and elasticity to the ECM. Fibulins assemble into microfibrils that bind with other components of the ECM. Fibulin-4 knockdown significantly decreased expression of E-cadherin and increased expression of N-cadherin, vimentin, Snail, Slug, and Twist. Endometrial fibroblasts also exhibited a significant decrease in vimentin and α-smooth muscle actin when co-cultured with fibulin-4 expressing endometrial epithelial cells (Wang et al., 2017). Fibulin-5 contains an RGD-motif, and mediates binding to integrin receptors α_4_β_1_, α_5_β_1_, α_9_β_5_, α_v_β_3_, and α_v_β_5_ (Yanagisawa et al., 2009). Fibulin-5 initiates EMT and is also a gene target of TGF-β1, indicated by Twist expression. In addition, fibulin-5 stimulated gelatinase proteolytic activity (Lee et al., 2008).

## 6. Commentary and Outlook

In this review, we have summarized work that highlights the important role of healthy epithelial ECM in maintaining epithelial polarization, while also investigating how fibrillar ECM drives EMT, with a focus on the roles of both soluble signals embedded in the ECM and the mechanical properties of the ECM. We have also discussed how the EMT process itself remodels the ECM to enable EMT progression.

While many aspects of the bidirectional regulation between the ECM and EMT signaling are well-understood, there are also key aspects of these signaling processes that remain to be fully elucidated. Although many individual pieces of these physiological puzzles have been studied in isolation, the emergent responses *in vivo* are inherently more complicated to understand. For example, which mechanochemical interactions that drive dynamics are critical for epithelial polarization and EMT, and which signaling pathways act in parallel and provide redundancy? Further, what are the mechanisms driving these individual interactions between mechanics and composition at the molecular and biophysical level? Additionally, what processes and features are cell type and/or species dependent, which is a critical question to understand ultimately what aspects of epithelial maintenance and EMT suppression can be critically translated? Studies that probe these questions represent the next steps forward in understanding the complex interactions between ECM and EMT.

## Author Contributions

LS, SW, and CL collectively formulated the structure and content of the review paper, wrote sections of the manuscript, and collectively edited the final manuscript.

### Conflict of Interest Statement

The authors declare that the research was conducted in the absence of any commercial or financial relationships that could be construed as a potential conflict of interest.
